# The Effect of a Best Practice Advisory on the Utilization and Impact of Palliative Care Consultation in Heart Failure Hospitalizations

**DOI:** 10.1089/pmr.2024.0106

**Published:** 2025-04-21

**Authors:** Kaitlyn S. Gooding, Vamsidhar V. Naraparaju, Beth Esstman, Dorothy B. Wakefield, Megan Evjen, Ahmed Naseer, Sara Tabtabai

**Affiliations:** ^1^ Department of Internal Medicine, University of Connecticut, Farmington, Connecticut, USA.; ^2^ Department of Cardiology, St Francis Hospital and Medical Center, Hartford, Connecticut, USA.; ^3^ University of Connecticut, Farmington, Connecticut, USA.; ^4^ Yale University School of Medicine, New Haven, Connecticut, USA.

**Keywords:** advanced heart failure, best practice advisory, heart failure, palliative care

## Abstract

**Background:**

Few studies examine palliative care consultations (PCC) in acute decompensated heart failure (ADHF) admissions. Prior data suggest that 6% of admitted patients are referred for PCC. This study evaluates the effect of a best practice alert (BPA) embedded in the electronic record on PCC utilization and outcomes.

**Methods:**

Patients admitted between May 1, 2020, and June 30, 2022, with ADHF were included. BPA was triggered at admission for patients with ≥3 ADHF admissions in 6 months or PCC during prior admission. Subjects were divided into early PCC (less than three days of admission), late PCC (more than three days), and no PCC. Demographics, BPA utilization, length of stay (LOS), and cost were compared between groups.

**Results:**

Of 684 patients, 18% received PCC: 13.1% had early PCC, 5.12% late PCC, and 81.6% no PCC. Early PCC patients were older with more comorbidities. Patients receiving PCC had lower ejection fraction (*p* = 0.04). Median LOS was longest in the late PCC group (12 days, *p* ≤ 0.01) and similar in early and no PCC groups (six and five days, respectively) and remained significant in multivariate analysis. White patients were more likely to receive PCC compared with Black and other races. The late PCC group had the lowest readmission rate at 5.7%; 28 of the 35 patients changed their goals of care to hospice, “do not re-hospitalize,” or “do not intubate/do not resuscitate.”

**Conclusions:**

PCC may influence therapy for patients with ADHF and reduce the readmission rate. Clinician biases remain despite the utilization of BPA, with a modest effect on PCC utilization.

## Background/Introduction 

Heart failure (HF) affects nearly 6.5 million Americans.^1^ It is the leading cause of hospitalizations in Medicare patients.^1,2^ The health care costs are staggering; projections estimate that HF admissions and treatment will exceed 70 billion dollars per year by 2033 (1). In many diagnoses, palliative care consultations (PCC) have been associated with reduced length of stay (LOS) and lower costs, with evidence that earlier PCC has improved cost benefit.^3–5^ Few studies have investigated the effect of PCC on patients admitted with HF.^6,7^

Organizations such as the American Heart Association and the American College of Cardiology recommend palliative care involvement in the treatment of HF patients.^8^ However, PCC remain underutilized in this patient population.^9,10^ There is a paucity of studies on the timing of PCC and the degree of benefit to patients and family members. In one of the few prior investigations of PCC in ADHF, only 6% of admitted patients were referred to palliative care^7^ even though each admission for acute decompensated heart failure (ADHF) has been shown to confer an increase in expected mortality.

In this study, we sought to describe ordering practices for PCC utilizing a best practice alert (BPA) embedded in the electronic record and correlate PCC with patient and health care utilization outcomes in a population of patients admitted with ADHF.

## Methods

**FIG. 1.**
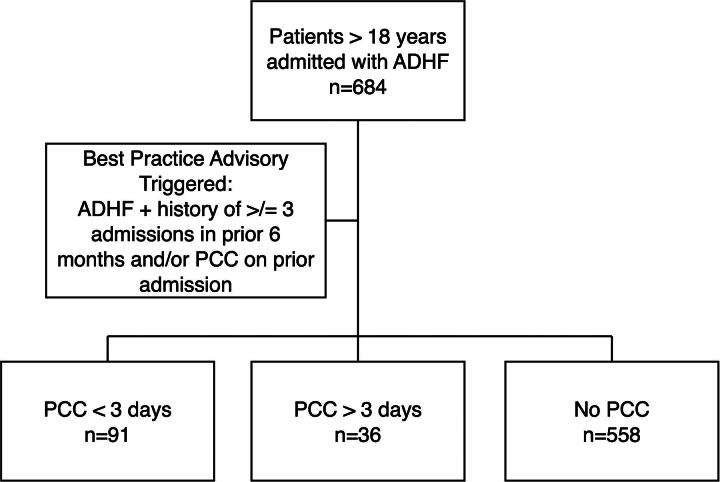
Flow chart of study design. ADHF, acute decompensated heart failure; PCC, palliative care consult.

The study design overview is shown in Figure 1. Patients admitted to our facility with the diagnosis of ADHF between May 1, 2020, and June 30, 2022, were identified and separated into three groups: patients who received an early PCC (defined as within three days of admission), patients who received a late PCC (defined as after three days of admission) and patients who did not receive PCC during their admission. A distinction of PCC within three days of admission was chosen for several reasons. First, prior work has noted that the magnitude of the effect of PCC on the cost of hospitalization is associated with the time from admission to the time of first consultation,^11^ thus establishing a time of consultation is preferred to having a binary “consult versus no consult” analysis. The timing of three days was chosen based on prior studies investigating multiple illnesses, including heart failure, performed within three days of admission reduced direct costs for hospitalized adults.^5,12^ Additional studies investigating PCC consultation during inpatient hospitalization have also used three days.^13–15^

Data were collected retrospectively from the electronic medical record (EMR). At our facility, a BPA has been integrated into the medical record to alert ordering clinicians to order a PCC at the time of admission for patients meeting either of the following criteria^1^: those admitted with ADHF and a history of *three or more* admissions for ADHF in the past 6 months, or^2^ patients with a PCC during a previous hospitalization in the past 12 months. The frequency with which the BPA was triggered was also recorded for each group and whether the consult was ordered.

Demographics for patients in each group were collected, including insurance status, race, ethnicity, age, sex, and language spoken. Hospital characteristics collected included LOS, charges, day of admission (weekday vs. weekend), and 30-day readmission rates. Clinical data and comorbidities were also collected and compared between groups. Categorical data were compared using chi-square analyses (age group, sex, race, ethnicity, insurance status, language spoken, weekend admission, 30-day readmission, BPA triggered, and comorbidities). Continuous data were compared using analysis of variance. Non-normal continuous data were compared using the Wilcoxon rank-sum test. Since LOS is a count variable, a negative binomial multivariable regression model examined the relationship between a PCC and LOS. Covariates considered were age group, sex, race, and comorbidities. Total hospital charges were log-transformed, and a multivariable linear regression examined the relationship between total charges and a PCC. Covariates considered were the same as above but also included LOS, as LOS is a large component of total charges. A backward selection model was performed to predict PCC that included age, race, EF, and all comorbidities that were significant in a bivariate case (diabetes, atrial fibrillation, coronary artery disease, pacemaker, renal insufficiency, depression, prior coronary artery bypass grafting, and TAVR). Statistical analyses were performed using SAS 9.4 (SAS Institute, Inc., Cary, NC, USA). The study was approved by the Trinity Health of New England Institutional Review Board.

## Results

**FIG. 2.**
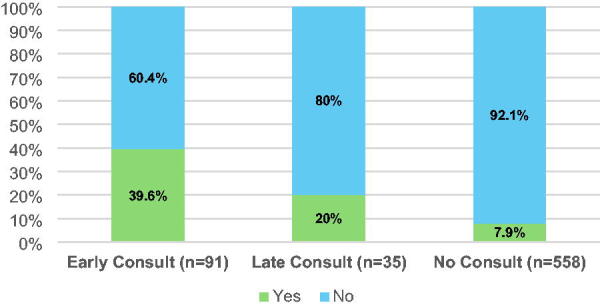
Percentage of best practice advisory triggered in palliative care consult groups.

A total of 684 patients admitted with a diagnosis of ADHF were included. Overall, 18% of patients received a PCC during admission. Specifically, 91 patients (13.3%) received an early PCC, 35 patients (5.12%) received a late PCC, and 558 patients (81.6%) did not receive a PCC during their hospitalization. Twenty-two patients had a PCC during a prior admission, all had a PCC during the study; 19 were in the early PCC group, and 3 were in the late PCC group. The baseline demographics, hospital characteristics, and clinical factors for each group are shown in Table 1. The BPA was triggered on admission in nearly 40% of the early PCC group, 20% of the late PCC group, and 8% of the group that received no PCC (Fig. 2). There was no difference in PCC if patients were admitted on a weekday or weekend. There were no in-hospital deaths recorded.

**Table 1.** Patient Demographics and Outcomes

	Early consult	Late consult	No consult	
	*n* = 91 (13.3%)	*n* = 35 (5.1%)	*n* = 558 (81.6%)	*p*-Value
Demographics				
Age, mean (SD)	81.2 (10.4)	75.7 (12.3)	73.5 (14.1)	**<0.01**
Age group, *n* (%)				**<0.01**
<65	10 (11)	7 (20)	148 (26.5)	
65–74	11 (12.1)	7 (20)	120 (21.5)	
75–84	31 (34.1)	10 (28.6)	154 (27.6)	
85 and up	39 (42.9)	11 (31.4)	136 (24.4)	
Sex, *n* (%)				0.70
Female	48 (52.8)	16 (45.7)	271 (48.6)	
Male	43 (47.3)	19 (54.3)	287 (51.4)	
Race, *n* (%)				**0.04**
Black	13 (14.3)	7 (20)	135 (24.2)	
White	72 (79.1)	26 (74.3)	354 (63.4)	
Other/unknown	6 (6.6)	2 (5.7)	69 (12.4)	
Hispanic ethnicity, *n* (%)	7 (7.7)	1 (2.9)	62 (11.1)	0.21
Insurance, *n* (%)				
Private	7 (7.7)	4 (11.4)	45 (8.1)	
Medicare	82 (90.1)	27 (77.1)	422 (75.6)	
Medicaid	2 (2.2)	4 (11.4)	86 (15.4)	
Self-pay	0 (0)	0 (0)	5 (0.9)	
Primary language, *n* (%)				0.55
English	80 (87.9)	31 (88.6)	474 (85)	
Spanish	5 (5.5)	0 (0)	28 (5)	
Other	6 (6.6)	4 (11.4)	56 (10)	
BPA triggered, *n* (%)	36 (39.6)	7 (20.0)	44 (7.9)	**0.01**
30-day readmission, *n* (%)	15 (16.5)	2 (5.7)	67 (12)	0.23
Weekend admission, *n* (%)	18 (19.8)	6 (17.1)	115 (20.6)	0.88
Outcome variables				
Hospital LOS, median (IQR)	6 (5, 9)	12 (7, 18)	5 (4, 8)	**<0.01**
Total charges (dollars), median (IQR)	40,340 (27,447, 62,910)	69,850 (43,361, 126,741)	37,126 (25,545, 55,673)	**<0.01**

Bold values are indicating significant *P* value of <0.05.

BPA, best practice alert; IQR, interquartile range; LOS, length of stay; SD, standard deviation.

A larger percentage of White patients received a PCC (early or late) compared with Black and other races. In fact, White patients were twice as likely to receive a PCC (early or late) as non-White patients (OR 95% CI: 2.02 (1.28–3.18). There was no difference in White versus non-White by sex and rates of BPA triggered. However, White patients had a higher average ejection fraction (EF) (47% vs. 43%, *p* = 0.02) and were older (65.5% of White patients were >75 years, while only 36.6% of non-White patients were >75 years, *p* < 0.01). Even when we controlled for age group and EF, White patients were still more likely to receive a PCC (*p* = 0.04). There was no difference in the likelihood of a PCC (early or late) by sex or primary language. White patients were more likely to have atrial fibrillation *(p* ≤ 0.0001), coronary artery disease *(p* = 0.04), a history of coronary artery bypass *(p* = 0.0008), a pacemaker (*p* = 0.01), and transcatheter aortic valve replacement (*p* = 0.0118). Non-white patients were more likely to have renal insufficiency (*p* = 0.0004) and diabetes mellitus (*p* = 0.003). White patients were 1.775 times more likely to have PCC when controlling for age group, EF, and renal insufficiency. Patients with renal insufficiency were 1.66 times more likely to have a PCC. No other comorbidities were significant predictors of PCC by race.

**FIG. 3.**
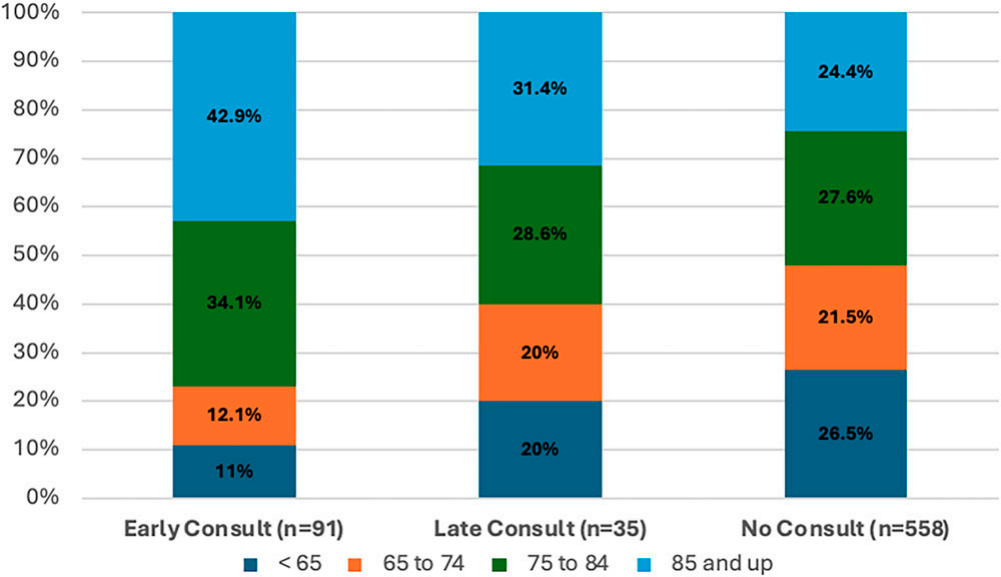
Timing of palliative care consult by age group.

PCC by age group is shown in Figure 3. The patients in the early consult group were older than both the late PCC and no-consult groups.

The baseline laboratory values, EF, and comorbidities of patients in each group are shown in Table 2. The patients receiving PCC had a lower EF than patients who did not receive a PCC (LVEF 43% vs. 47%, *p* = 0.04). The patients in the early PCC group were also more likely to have comorbidities including atrial fibrillation, hypertension, anemia, and renal insufficiency. In contrast, a larger percentage of patients in the late PCC group had valvular heart disease.

**Table 2.** Differences in Laboratory Values and Comorbidities by PCC Group

	Early consult	Late consult	No consult	*p*-Value
	*n* = 91 (13.3%)	*n* = 35 (5.1%)	*n* = 558 (81.6%)	
Lab results at admission				
HgB, mean (SD)	11.37 (1.95)	10.96 (2.03)	11.14 (2.04)	0.51
BNP, mean (SD)	1085.85 (924.72)	974.97 (877.91)	912.28 (818.56)	0.18
Serum creatinine, mean (SD)	1.55 (0.87)	1.55 (1.13)	1.49 (0.86)	0.76
EF%, mean (SD)	42.78 (19.04)	42.91 (18.68)	46.57 (16.08)	0.07
eGFR (at discharge), mean (SD)	45.67 (14.34)	46.71 (14.59)	46.99 (14.11)	0.72
Comorbidities				
CMI, mean (SD)	1.47 (0.51)	1.52 (0.58)	1.53 (0.89)	0.38
Afib, *n* (%)	54 (59.3)	19 (54.3)	235 (42.1)	**<0.01**
COPD/asthma, *n* (%)	39 (42.9)	14 (40)	181 (32.4)	0.12
Hyperlipidemia, *n* (%)	69 (75.8)	24 (68.6)	378 (67.7)	0.30
HTN, *n* (%)	83 (91.2)	26 (74.3)	500 (89.6)	**0.01**
Peripheral vascular disease, *n* (%)	12 (13.2)	5 (14.3)	60 (10.8)	0.67
CAD, *n* (%)	44 (48.4)	15 (42.9)	222 (39.8)	0.30
CVA/TIA, *n* (%)	20 (22)	6 (17.1)	77 (13.8)	0.12
ICD only, *n* (%)	8 (8.8)	4 (11.4)	34 (6.1)	0.33
Anemia, *n* (%)	40 (44.0)	11 (31.4)	152 (27.2)	**<0.01**
Pacemaker, *n* (%)	14 (15.4)	5 (14.3)	46 (8.2)	0.06
Renal insufficiency, *n* (%)	34 (37.4)	5 (14.3)	125 (22.4)	**<0.01**
Depression, *n* (%)	27 (29.7)	7 (20)	146 (26.2)	0.53
Valvular heart disease, *n* (%)	16 (17.6)	10 (28.6)	61 (10.9)	**<0.01**
Sleep-disordered breathing, *n* (%)	23 (25.3)	8 (22.9)	176 (31.5)	0.30
DM, *n* (%)	40 (44)	13 (37.1)	285 (51.1)	0.15
Total comorbidities, median (IQR)	7 (5, 8)	6 (4, 8)	6 (4, 7)	**<0.01**
Prior PCI, *n* (%)	13 (14.3)	3 (8.6)	85 (15.2)	0.55
Prior CABG, *n* (%)	19 (20.9)	6 (17.1)	100 (17.9)	0.78
Prior MI, *n* (%)	25 (27.5)	8 (22.9)	105 (18.8)	0.15

BNP, B-type natriuretic peptide; CABG, coronary artery bypass grafting; COPD, chronic obstructive pulmonary disease; CVA/TIA, cerebrovascular accident / transient ischemic attack; DM, diabetes mellitus; EF, ejection fraction; HgB, hemoglobin; eGFR, estimated glomerular filtration rate; ICD, Implantable cardioverter defibrillator; MI, myocardial infarction; PCI, percutaneous coronary intervention.

There was a significant difference in LOS between groups. The median LOS was the longest in the late PCC group (12 days, *p* ≤ 0.01). The median LOS was similar in the early PCC and no PCC groups (six and five days, respectively). Multivariable analysis indicated that the difference in LOS between PCC groups remained significant even when controlling for covariates (Table 3). Compared with patients who did not have a PCC consult, early consult patients had 1.25 times the number of hospital days, and late consult patients had 2.25 times the number of hospital days. Patients aged 85 and older had a significantly shorter LOS compared with those in the less than 65-year-old group (*p* < 0.01, relative risk [95%]: 0.73 [0.65, 0.83]). There was no significant difference in LOS for patients who had a PCC between those with and without a PCC on a prior admission.

**Table 3.** Negative Binomial Regression and Relative Rates of Length of Stay

	Estimate (SE)	*p*-Value	Relative rate (95% CI)
Study group	** **	**<0.01**	** **
Early consult	0.22 (0.06)	**<0.01**	1.25 (1.11, 1.41)
Late consult	0.81 (0.08)	**<0.01**	2.25 (1.9, 2.65)
No consult	ref		
Age group		**<0.01**	
85+	−0.31 (0.06)	**<0.01**	0.73 (0.65, 0.83)
75–84	−0.1 (0.06)	0.10	0.91 (0.81, 1.02)
65–74	−0.05 (0.06)	0.39	0.95 (0.84, 1.07)
<65	ref		
Hypertension, Y vs. N	0.2 (0.07)	**<0.01**	1.22 (1.07, 1.4)
CAD, Y vs. N	−0.11 (0.05)	**0.03**	0.89 (0.81, 0.99)
ICD only, Y vs. N	−0.21 (0.09)	**0.02**	0.81 (0.68, 0.96)
Pacemaker, Y vs. N	0.14 (0.07)	0.06	1.15 (0.99, 1.33)
Prior CABG, Y vs. N	0.14 (0.06)	**0.03**	1.15 (1.01, 1.31)
Sleep-disordered breathing, Y vs. N	0.15 (0.05)	**<0.01**	1.16 (1.06, 1.27)

CI, confidence interval; SE, standard error.

Total charges for hospital stays were different between PCC groups (*p* < 0.01). Charges were highest among the late consult group due to longer LOS. In contrast, multivariable analysis that controlled for LOS, case mix index, and sleep-disordered breathing found no difference in total hospital charges by the PCC group (*p* = 0.99, Table 4).

**Table 4.** Linear Regression Predicting Log-Transformed Hospital Charges

Parameter	Estimate (SE)	*p*-Value
Study group	** **	0.99
Early consult	0.003 (0.036)	
Late consult	−0.004 (0.059)	
No consult	ref	
Length of stay	0.096 (0.003)	**<0.01**
Case mix index	0.142 (0.016)	**<0.01**
Sleep-disordered breathing (yes vs. no)	0.07 (0.026)	**<0.01**

There was no significant difference in 30-day readmissions between groups (*p* = 0.23). Early PCC had a readmission rate of 16.5%, late PCC 5.7%, and no consult 12%. Due to the large discrepancy between readmission rates, further chart review of the late PCC group was performed. Of the patients in the late PCC group, 8 patients changed their goals of care to hospice or “do not re-hospitalize,” and 20 patients changed their code status to “do not intubate/do not resuscitate.”

## Discussion

In this study, we sought to describe the impact of a BPA on PCC in patients hospitalized with ADHF and the effect of the timing of palliative care intervention on outcomes. There is a relative paucity of literature on palliative care in the HF population, especially during an acute hospitalization. To our knowledge, this represents one of the few studies investigating ways to involve palliative care, what time course of involvement in an acute hospitalization may be of benefit, and the association of PCC involvement and outcomes. In one of the few prior investigations of PCC in ADHF, only 6% of admitted patients were referred to palliative care.**^7^** We found that the use of a BPA tool embedded in the EHR resulted in a modest increase in PCC compared with this historical figure. In the late PCC group, there was a high rate of change in goals of care, and there was a trend toward the lowest readmission rate. Finally, we found demographic and clinical differences in patients with early PCC, which may represent clinician bias.

We found that LOS was significantly different between the groups that did have PCC despite a relatively small sample size in these groups. Interestingly, the LOS for the no PCC group was like the early PCC group. In the no PCC group, the BPA was triggered for only 8% of the patients. Given that the BPA was triggered for patients that had three or more admissions in the past 6 months or PCC on a prior hospitalization in the past 12 months, this may suggest a lower-risk subset of HF patients in the no PCC group. The BPA fired in the early PCC consult 40% of the time, suggesting a higher risk group. The difference in LOS between the groups persisted when correcting for other factors in a multivariate analysis. Our data suggest that early PCC may decrease LOS akin to that of a lower-risk ADHF population. The finding of lower LOS is in line with studies investigating PCC involvement in inpatient admissions of other diagnoses that show decreased LOS.^5,13–15^ To our knowledge, this is the first report on the effect of PCC on LOS in patients admitted with ADHF.

The late PCC group had a trend toward the lowest 30-day readmission rate. There is a known inverse association between LOS and 30-day readmission rates.^16^ However, in our study, due to the surprisingly low number of readmissions in the late PCC group, further investigation revealed that goals of care and code status were changed during the index hospitalization in 80% of the patients in this group. After the involvement of the palliative care team, 8 patients were transitioned to hospice or “do not re-hospitalize,” and 20 patients were transitioned from “full code” to “do not resuscitate/do not intubate.” These findings are hypothesis-generating and suggest that involving palliative care in ADHF admissions may influence a patient’s choice of therapy and ultimately lead to decreased readmission rates.

We found demographic and clinical differences in patients who received early PCC. Older patients were more likely to have a PCC ordered within three days of admission. As there was no age trigger for BPA in our study, this may reflect both the increased likelihood of comorbidities in the older group as well as preconceived notions that older patients have an increased risk of death in HF. Patients with a lower EF were more likely to be in the early PCC group. Prior studies have shown that heart failure with preserved EF (HFpEF) tends to have a less recognized risk of severity of illness and mortality.^17^ We have also shown in prior work that HFpEF is less likely to be recognized as the primary cause of admission or coded as the primary diagnosis.^18^ Our findings highlight an increased likelihood to involve palliative care when patients are older and EF is lower. Further studies and education are warranted to consider PCC across the spectrum of age and EF.

White patients were more likely to receive early PCC in our study. There is limited research on racial and ethnic disparities in palliative care utilization and outcomes, especially in chronic diseases other than cancer.^19^ Our data are in line with a prior study using the Nationwide Inpatient Sample, which found that being of a racial and ethnic minority predicted lower odds of having an encounter with palliative care during an HF admission.^20^ The White patients in our study who received PCC were older compared with non-White groups. However, even when controlling for age, White patients were still more likely to receive PCC during their admission. This data further support avoiding age as a trigger for the involvement of palliative services in a patient’s care. Racial minority groups are likely underrepresented in the elderly HF population, as currently only 24% of older adults are racial or ethnic minorities.^21^ Additionally, Black and Hispanic patients are more likely than White patients to have a younger age of HF onset.^20^ The Coronary Artery Risk Development in Young Adults study described a 20-fold higher incidence of HF in young Black patients under the age of 50.^22^ Age-standardized hospitalization rates and age-adjusted 30-day case fatality rates are higher for Black patients.^21^ Therefore, using age as a trigger for HF risk and PCC is likely to underrecognize the risk and severity of illness in minority HF patients. Unfortunately, even when controlling for age and other comorbidities in our study, we found a discrepancy in palliative involvement by race.

White patients in our study were also more likely to have higher EF, at 47% compared with 43%. As we have previously discussed, patients with a lower EF are more likely to be recognized as at risk for poor outcomes. Despite having a lower EF, non-White patients were not referred for palliative care as frequently as White patients in this study. Further studies are needed on the involvement of palliative care in minority groups, including how the increased proportion of elderly minorities in the future may be affected and the recognition of disease severity by common measures such as EF may be confounded or underrecognized.

## Limitations

There are inherent limitations to this study, as it was a single-center, retrospective chart review and not a randomized controlled trial. In the absence of a randomized study, this study can only comment on the correlation between timing and use of PCC with patient outcomes and utilization factors, including LOS, and not necessarily causation. There are inherent confounders that likely influenced the LOS and cost. We attempted to account for these by utilizing multivariate models.

This study also did not have information on why PCC may not have been ordered when BPA was triggered and whether PCC was declined by the patient. Future multicenter studies with a randomized design of PCC for patients admitted with ADHF that also collect provider and patient insights on the involvement of palliative care in their multidisciplinary team will help clarify the points raised by this study.

Finally, this study coincided with the peak of the COVID-19 pandemic. The effect of the pandemic on care delivery patterns and patient outcomes in this study cannot be excluded.

## Conclusions

To our knowledge, this is one of the only studies to focus specifically on the effect of PCC on patients admitted with ADHF and the role of a BPA to augment PCC. A BPA resulted in a modest increase in PCC compared with historical values. PCC may influence the choice of therapy in patients hospitalized with ADHF and, in so doing, reduce the readmission rate. Clinician biases remain despite the utilization of electronic health record-embedded tools and had only a modest effect on PCC ordering.

## Abbreviations Used

ADHF: acute decompensated heart failure
BPA: best practice alert
EF: ejection fraction
HF: heart failure
LOS: length of stay
PCC: palliative care consultations
TAVR: transcatheter aortic valve replacement
